# A long-term survival case after resection of the pancreatic metastasis from lung cancer

**DOI:** 10.1016/j.ijscr.2019.07.060

**Published:** 2019-07-25

**Authors:** Yumiko Kageyama, Ryuzo Yamaguchi, Shinya Watanabe, Keiji Aizu, Fumiya Sato, Hironori Fujieda, Mihoko Yamada, Yoshitaka Toyoda, Tsutomu Iwata

**Affiliations:** Division of Surgery, Kasugai Municipal Hospital, Aichi, Japan

**Keywords:** CEA, carcinoembryonic antigen, CT, computed tomography, PET, positron emission tomography, Pancreatic metastasis, Lung cancer, Surgical resection, Combined therapy

## Abstract

**Introduction:**

Pancreatic metastasis from lung cancer is not an indication for surgical resection because patients with such a condition present with multiple-organ metastases. Therefore, the significance of resection in patients with pancreatic metastasis from lung cancer remains unclear. Here we report a case of a long-term survivor of pancreatic metastasis from lung cancer after pancreatectomy.

**Case presentation:**

A 67-year-old woman presented with a pancreatic mass. She had undergone left lower lobectomy for stage IIIA lung adenocarcinoma 6 years prior to presentation. Following surgery, she received adjuvant treatment with gefitinib for 7 months. However, this treatment was discontinued due to its side effects. The patient received radiation therapy for mediastinal lymph node metastasis 2 years after resection and she became cancer-free. Six years after the initial pulmonary resection, the patient’s tumor marker level increased, and abdominal computed tomography (CT) revealed a 20-mm tumor in the pancreatic tail. Positron emission tomography–CT revealed an abnormal uptake in the pancreatic tail. However, no other abnormal lesions were observed. The diagnosis was primary pancreatic cancer or metastasis from lung cancer. Distal pancreatectomy with lymph node dissection was performed, and the pathological diagnosis was metastasis from lung cancer. The patient survived for more than 5 years without recurrence but she died of acute renal failure after acquiring pneumonia.

**Conclusion:**

Surgical treatment should be considered for pancreatic metastasis from lung cancer if the disease is localized and the patient’s condition is good. Additionally, combined therapy, including surgical resection, may be effective for repeated recurrence.

## Highlights

• Significance of resection in patients with pancreatic metastasis from lung cancer.
• A long-term survivor of pancreatic metastasis from lung cancer after pancreatectomy.
• Consider resection if the disease is localized and the patient’s condition is good.

## 1 Introduction

The impact of surgical resection of limited metastatic tumor, such as liver or lung metastasis, has been clearly defined. However, the benefits of surgical resection in patients with pancreatic metastasis remains unclear because pancreatic metastasis is rarely resected given that most patients present with additional organ metastases [1,2]. This study reports the case of a cancer survivor who lived for more than 5 years after the resection of pancreatic metastatic tumors from lung cancer and present a literature review to discuss the significance of surgical resection in such patients. This work has been reported in line with the SCARE criteria [3].

## 2 Case presentation

**Fig. 1:**
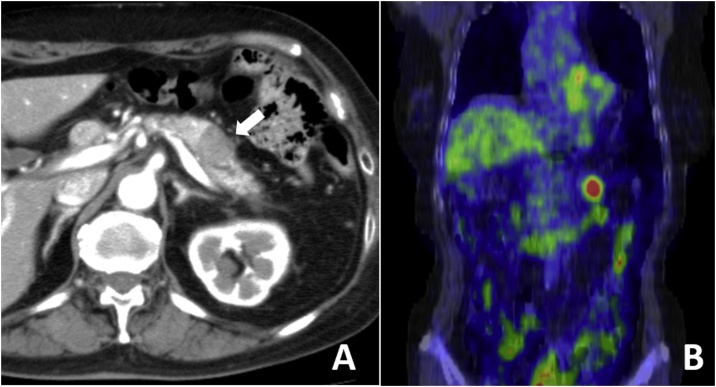
Preoperative findings. **(A)** Computed tomography reveals a low density mass in the pancreatic tail *(allow).* **(B)** Positron emission tomography shows a hypermetabolic mass in the pancreatic tail.

**Fig. 2:**
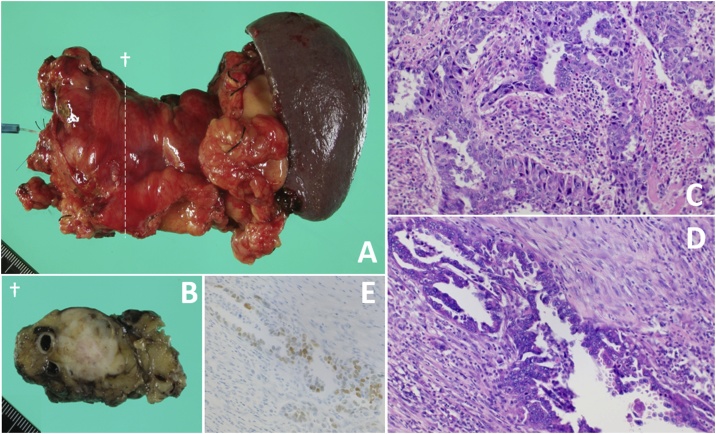
Resected specimen. **(A, B)** A 25 × 25-mm tumor was located in the pancreatic tail. **(C)** Histological findings of the lung tumor showing adenocarcinoma. **(D)** Histological findings of the pancreatic tumor showing adenocarcinoma. **(E)** The pancreatic tumor was TTF-1 positive, like that of the resected primary lung cancer.

A 67-year-old woman presented with a mass located in the pancreatic tail. The patient’s past medical history included pneumonia and lung cancer. Six years prior to presentation, she had undergone left lower lobectomy with systematic lymphadenectomy for stage IIIA adenocarcinoma. After resection, she received adjuvant gefitinib for 7 months; however, this treatment was discontinued due to side effects. Two years after resection, the patient received radiation therapy for mediastinal lymph node metastasis and she eventually became cancer-free. Two years after radiation therapy, the patient’s carcinoembryonic antigen (CEA) level was elevated at 13.5 U/mL, and computed tomography (CT) revealed a low-density mass, which measured 20 mm in diameter, in the pancreatic tail (Fig. 1A). Positron emission tomography (PET)/CT revealed a hypermetabolic mass in the pancreatic tail, with no other distant metastases (Fig. 1B). Primary pancreatic cancer or metastasis from lung cancer was suspected. The tumor was localized in the pancreas and the patient’s general status was good; therefore, distal pancreatectomy and splenectomy with lymph node dissection were performed for curative intent. The resected specimen from the pancreatic tail was a 25 × 25-mm tumor (Fig. 2A, B). The pathological diagnosis was metastatic lung adenocarcinoma (Fig. 2C, D), with immunohistochemical profiles (cytokeratin 7- and thyroid transcription factor 1-positive) similar to those of the resected primary lung cancer (Fig. 2E). The postoperative course was uneventful. The patient survived for 5 years and 3 months after pancreatectomy, and no evidence of recurrence was noted. However, she died of acute renal failure after acquiring pneumonia.

## 3 Discussion

The pancreas is an unusual site for metastasis. Pancreatic metastasis accounts for approximately 1%–5% of all pancreatic malignant diseases [2,[4], [5], [6], [7]]. Conversely, the most common primary tumors of pancreatic metastasis include renal cell carcinoma, followed by lung and colorectal tumors [8]. Moreover, pancreatic metastasis has been reported in up to 18% of patients with lung cancer [9,10], thus suggesting that pancreatic metastasis from lung cancer is not extremely rare. However, only few patients are qualified for resection because most patients with lung cancer present with widespread diseases in multiple organs [10]. Therefore, the importance of surgical resection in patients with pancreatic metastasis from lung cancer is inadequately evaluated in the literature.

The most common diagnostic examination for pancreatic metastasis is annual examination, which includes radiological imaging or serum tumor marker level assessment s during the treatment of lung cancer. Other patients present with obstructive jaundice and acute pancreatitis [11]. In our study, PET/CT was immediately performed when the patient presented with an elevated CEA level, which was useful for detecting metastatic lesion and evaluating surgical indication.

The differential diagnosis between primary and metastatic pancreatic tumors on the basis of CT, endoscopic retrograde cholangiopancreatography, or ultrasonography is challenging [12,13]. In our study, we could not distinguish the primary pancreatic tumor from the metastatic pancreatic tumor preoperatively. Therefore, we decided to perform curative resection at least for the primary tumor. We believe that even if patients present with pancreatic metastasis, resection can also be performed for curative intent; however, the significance of survival benefit remains uncertain.

We conducted a review of the literature, which includes Japanese studies, by searching PubMed, and identified 22 reports on aggressive surgical resection for both primary lung cancer and pancreatic metastasis. We assessed 23 cases, including the case in the present study. The following cases were observed: n = 10, histopathologically confirmed adenocarcinoma; n = 6, squamous cell carcinoma; n = 3, large-cell carcinoma; n = 2 adenosquamous carcinoma; and n = 2, small-cell carcinoma. Only 2 cases involved synchronous metastases, whereas the remaining 21 cases were metachronous. Moreover, the median interval time from pulmonary resection to pancreatic recurrence was 22 months. Patient survival for more than 5 years after metastatic pancreatic tumor resection was only reported in the study by Ida [14] and in our study. Ida reported that pancreatic metastasis was diagnosed in the patient 3 years after pneumonectomy for lung squamous cell carcinoma. This patient underwent total pancreatectomy and survived for more than 8 years without recurrence. Conversely, the patient in our study received combined therapy, which includes chemotherapy after the resection of primary tumor, radiation for lymph node metastasis, and resection of metastatic pancreatic tumor. In our literature review, no specific clinicopathological features were observed in long-term survivors.

Iida et al. [15] have reported that the mean survival time in patients with pancreatic metastasis from lung cancer without pancreatectomy is 6.3 months. Although the indication for pancreatectomy for pancreatic metastasis from lung cancer cannot be defined based on only two long-term survivors, we believe that radical treatment, including pancreatectomy, results in the marked improvement of prognosis in some cases. Further studies discussing treatment options, including surgical resection, for pancreatic metastasis from lung cancer are warranted.

## 4 Conclusion

If the disease is localized and the patient’s condition is good, aggressive resection for pancreatic metastasis from lung cancer should be considered. In the present case, combined therapy, including surgical resection, resulted in long-term survival, and the patient was declared cancer-free despite repeated cancer recurrence.

## Funding

This research received no specific grant from any funding agency in the public, commercial, or not-for-profit sectors.

## Ethical approval

The ethical approval has been exempted by our institution because this is a case report.

## Consent

Written informed consent was obtained from the patient for publication of this case report and any accompanying images. A copy of the written consent is available for review by the Editor-in-Chief of this journal upon request.

## Author contribution

YK prepared the draft of the report and collected data. RY supervised the treatment of the patient and edited the manuscript. SW and KA assisted in preparing the manuscript. FS, HF, MY, YT and TI contributed to drafting the manuscript. All authors have read and approved the final manuscript.

## Registration of research studies

Not applicable.

## Guarantor

Yumiko Kageyama and Ryuzo Yamaguchi

## Provenance and peer review

Not commissioned, externally peer-reviewed

## Declaration of Competing Interest

No conflict of interest.

## References

[1] Hiotis S.P., Klimstra D.S., Conlon K.C., Brennan M.F. (2002). Results after pancreatic resection for metastatic lesions. Ann. Surg. Oncol..

[2] Roland C.F., van Heerden J.A. (2009). Nonpancreatic primary tumors with metastasis to the pancreas. The role of surgery management of isolated metastases to the pancreas. Lancet Oncol..

[3] Agha R.A., Borrelli M.R., Farwana R., Koshy K., Fowler A., Orgill D.P., For the SCARE Group. The SCARE (2018). Statement: Updating Consensus Surgical CAse REport (SCARE) Guidelines. Int. J. Surg..

[4] Adsay N.V., Andea A., Basturk O., Kilinc N., Nassar H., Cheng J.D. (2004). Secondary tumors of the pancreas: an analysis of a surgical and autopsy database and review of the literature. Virchows Arch..

[5] Z’Graggen K., Fernandez-del Castillo C., Rattner D.W., Sigala H., Warshaw A.L. (1998). Metastases to the pancreas and their surgical extirpation. Arch Surg..

[6] Crippa S., Angelini C., Mussi C., Bonardi C., Romano F., Sartori P. (2006). Surgical treatment of metastatic tumors to the pancreas: a single center experience and review of the literature. World J. Surg..

[7] Zerbi A., Ortolano E., Balzano G., Borri A., Beneduce A.A., Di Carlo V. (2008). Pancreatic metastasis from renal cell carcinoma: which patients benefit from surgical resection?. Ann. Surg. Oncol..

[8] Rumancik W.M., Megibow A.J., Bosniak M.A., Hilton S. (1984). Metastatic disease to the pancreas: evaluation by computed tomography. J. Comput. Assist. Tomogr..

[9] Abrams H.L., Spiro R., Goldstein N. (1950). Metastases in carcinoma: analysis of 1000 autopsied cases. Cancer.

[10] Maeno T., Satoh H., Ishikawa H., Yamashita Y.T., Naito T., Fujiwara M. (1998). Patterns of pancreatic metastasis from lung cancer. Anticancer Res..

[11] Notake T., Nakayama A., Takeuchi N., Tujimoto K., Ito N., Takasuna K. (2011). A case of pancreatic metastasis from small cell carcinoma of the lung. J Jpn Surg Assoc..

[12] Swensen T., Osnes M., Serck-Hanssen A. (1980). Endoscopic retrograde cholangio-pancreatography in primary and secondary tumors of the pancreas. Br. J. Radiol..

[13] Wernecke K., Peters P.E., Galanski M. (1986). Pancreatic metastases: US evaluation. Radiology.

[14] Ida T., Yoshida M., Naito K., Iitsuka K. (2006). An eight-year survivor after the resection of a metastatic pancreatic tumor of pulmonary carcinoma. J Jpn Surg Assoc..

[15] Iida T., Sekoguchi T., Yamamoto T., Nakamura K., Ito F., Sakurai H. (2002). A case of resected metastatic pancreatic tumor from lung cancer complicated by acute pancreatitis. J Jpn Panc Soc..

